# Routine gynecological ultrasound: look at the bladder and the ureters!

**Published:** 2020-03-27

**Authors:** M Vangoitsenhoven, V Vandenbroucke, T Van Den Bosch

**Affiliations:** Department of Obstetrics & Gynecology, RZ Tienen, Belgium;; Department of Obstetrics & Gynecology, University Hospital Leuven, Belgium.

**Keywords:** transvaginal ultrasonography, ureters, bladder, urinary tract

## Abstract

**Background:**

To illustrate the technical feasibility and diagnostic value of including the assessment of the bladder and the ureters during a standard transvaginal ultrasound examination.

**Methods:**

We present four cases illustrating ureter and bladder pathology diagnosed by transvaginal ultrasound.

**Results:**

In a first case, transvaginal ultrasonography in a woman with lower abdominal pain showed a calculus in the left distal ureter. The small echogenic lesion was easily detectable within the ureter lumen. A second patient, presenting with suprapubic pain, urgency and back pain, was also diagnosed with a ureteral calculus and additionally a hydroureter. The presence of a hydroureter is part of the differential diagnosis of any cystic structure in the pelvis. In a third case, an elderly woman, referred with uterine prolapse, transvaginal ultrasound examination showed a tubular anechoic structure lateral in the pelvis due to a hydroureter, illustrating the differential diagnosis with a hydrosalpinx. A fourth patient, presenting with hematuria, showed an irregular and highly vascularized mass in the bladder and was diagnosed with a transitional cell carcinoma.

**Conclusion:**

The bladder and pelvic part of the ureters are easily identifiable on transvaginal scan. Important pathology of the lower urinary tract and the distal ureters can be readily diagnosed by transvaginal ultrasound examination. The evaluation of the bladder and the ureters should therefore be part of the standard gynecological ultrasound investigation.

## Introduction

Gynecological ultrasound assessment performed at the gynecology outpatient clinic or at the emergency department is often limited to the evaluation of the uterus and the ovaries. However, in the last decade, the introduction of ultrasound examination in urogynecology (Dietz, 2019) and in the diagnostic workup of deep endometriosis (Pateman et al. 2015; Guerriero et al., 2016) showed that the anterior compartment of the pelvis is easy to evaluate by transvaginal and perineal ultrasound.

Gynecological ultrasound examination is often performed in case of abdominal pain or abnormal bleeding. Besides gynecological pathology, the differential diagnosis includes urological conditions such as urolithiasis, infection or malignancy. Transvaginal ultrasound is readily available, cheap and quickly performed. Because the vaginal probe can be placed in the immediate vicinity of the bladder, the urethra or the distal ureters, the image is not hampered by overlying tissue, contrary to transabdominal scanning. As opposed to CT-scan or MRI, applying gentle pressure with the transvaginal probe, site-specific tenderness can be assessed, often guiding the examiner to the area of interest.

The aim of our paper is to illustrate the feasibility and diagnostic value of including the evaluation of the bladder and the ureters during routine transvaginal ultrasound examination.

## Methods

To emphasize the importance of including the evaluation of the anterior pelvic compartment in routine gynecological scanning,we present a case series illustrating ureter and bladder pathology diagnosed by transvaginal ultrasound. Chronic conditions such as deep endometriosis or uro- gynecological pathology, such as a cystocoele, are beyond the scope of this paper. We focus on imaging technique, while mentioning some tips and tricks and discussing some pitfalls.

## Results

### Case 1

**Figure 1:**
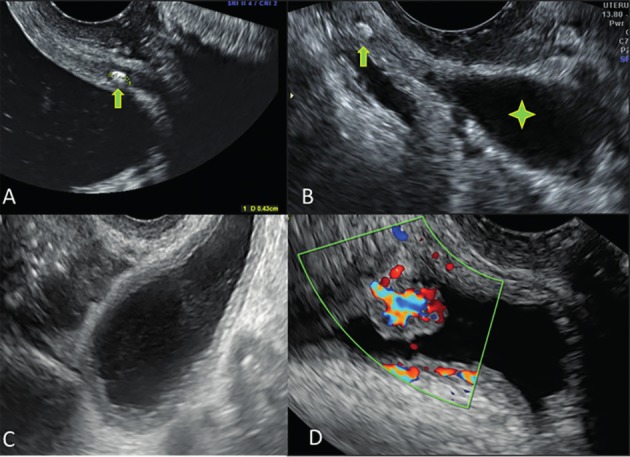
Transvaginal ultrasound images illustrating urological pathology. A: calculus (arrow) in the distal part of the ureter. B: calculus in the distal part of the ureter (arrow) with proximal dilatation of the ureter (star). C: hydro-ureter mimicking an adnexal mass. D: highly vascularized malignant lesion in the bladder wall.

A 29-year-old nulliparous patient presented to the emergency department with continuous pain in the left iliac fossa, slightly irradiating to the left lumbar region. She had one episode of vomiting the day before. Transabdominal ultrasound examination did not show any significant abnormalities. The bladder was empty and could not be evaluated. She was referred to the gynecology department to rule out any gynecological emergency such as an ovarian torsion. Transvaginal ultrasound examination showed a small, 4mm diameter, hyperechogenic calculus within the left distal ureter lumen, at 18mm from the ureteral ostium (Figure 1A).

### Case 2

A 41-year-old patient presented at the gynecology outpatient clinic with urgency, suprapubic pain and occasional right backpain in the last two weeks. Vaginal ultrasound examination revealed a 4mm diameter hyperechogenic distal right ureteric calculus. Cranially from the lithiasis, the ureter showed a progressive dilatation up to 25mm diameter (Figure 1B). Abdominal ultrasound examination showed that the hydroureter was associated with a mild hydronephrosis of the right kidney.

### Case 3

A 79-year-old woman (P2G2) was referred to the gynecology outpatient clinic for uterine prolapse. On vaginal ultrasound examination, a tubular thick- walled cystic structure with anechoic content was seen left of the uterus (Figure 1C). The differential diagnosis between a left-sided hydrosalpinx, an cystic ovarian mass and a prominent hydroureter was made by more detailed examination of the location of the cystic lesion and the bladder wall. Abdominal ultrasound examination showed massive hydronephrosis, almost without visible residual renal cortex.

### Case 4

A 74-year-old nulliparous woman presented at the gynecology outpatient clinic with hematuria since one month. According to the patient, a urological examination one month before, including a transabdominal scan, could not evidence any lesion. Vaginal ultrasonography showed an irregular, strongly vascularized lesion of 10x8mm protruding into the bladder cavity positioned cranially to the urethra (Figure 1D). Cystoscopic biopsy confirmed the sonographic suspicion of a transitional cell carcinoma.

## Discussion

The presented cases illustrate the importance of the evaluation of the anterior compartment of the pelvis during ‘gynecological’ ultrasound assessment. We believe that gynaecological ultrasound training should include the teaching of the normal anatomy of the structures surrounding the uterus allowing to make the right differential diagnosis, cope with incidental findings, and choose the most appropriate management.

The urethra, the bladder and the distal part of the ureters are easily accessible by transvaginal ultrasound. As soon as the bladder shows minimal filling, the anechogenic bladder content is visible on ultrasound. To differentiate a distended bladder from a large anechogenic (ovarian) cyst, the urethra is visualized at the most caudal part in the midsagittal plane, ventral of the lower anterior vaginal wall.

The urethra is also a reference point in urogynecology to assess urethral hypermobility during Valsalva manoeuvre (Dietz, 2016). Gentle pressure on the urethra may cause site-specific tenderness in case of urethritis.

The trigonum is delineated by the urethra and both ureteric orifices; it is localized ventral to the anterior fornix of the vagina. Site-specific tenderness may be present in case of cystitis or deep vesicouterine endometriosis (Guerriero et al., 2016). The bladder base is the level where a small cystocoele may be evidenced during Valsalva.

**Figure 2:**
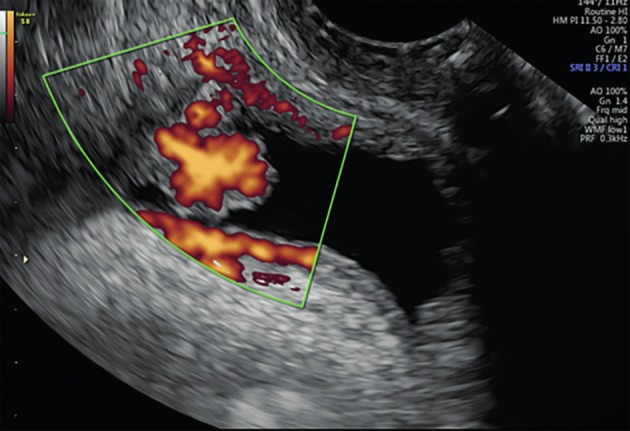
Transvaginal sonography displaying a highly vascularized malignant lesion in the bladderwall.

The bladder walls are generally regular, especially if the bladder is full. Irregular, highly vascularized protrusions into the bladder lumen are highly suspicious for transitional cell carcinoma (TCC) (Betsas et al., 2008) (Figure 2), while deep endometriosis lesions are usually somewhat less irregular and less vascularized (Pateman et al., 2015; Guerriero et al., 2016).

Especially in elderly women, the differentiation between vaginal/uterine bleeding and hematuria may be difficult to deduce from the patient’s history. In case of urinary incontinence, reported bloodstained underclothing may as well be from urological as from gynecological origin.

**Figure 3:**
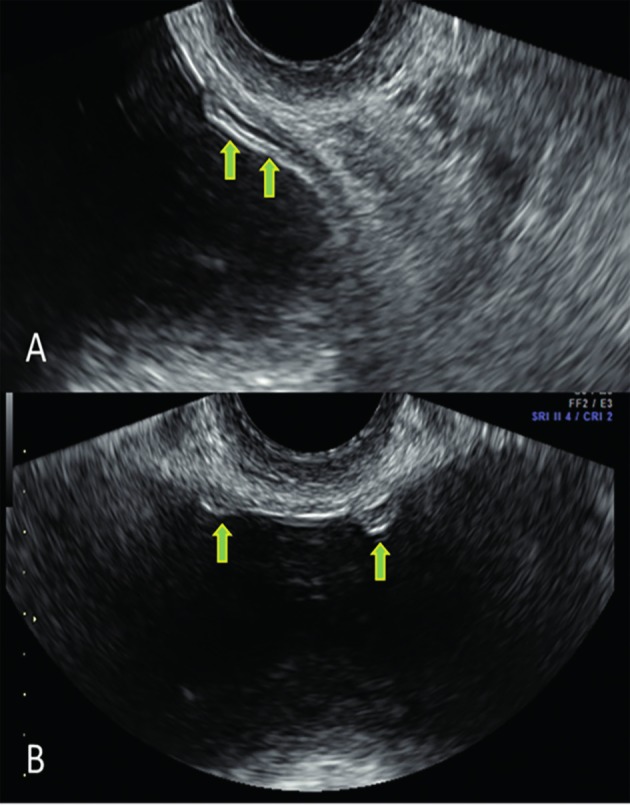
Distal part ot the ureter crossing the bladder wall (note the presence of some anechoic urine in the ureter lumen and the ureter wall). B: both ureteral orifices in transverse section

The ureter can be seen at vaginal ultrasonography as a thin relative hypoechogenic stripe tangential to the posterolateral bladder wall (Bean, 2018), typically with a regular thin anechoic (Figure 3A) or echogenic midline echo. To find the ureter, we usually start from a sagittal plane through the urethra, and subsequently move the vaginal probe laterally while rotating the probe slightly towards the median line (i.e. slightly anti-clockwise for the left ureter and clockwise for the right ureter).

Alternatively, to find the orifices of the ureters, we scan in the transverse plane between the urethra and the lower posterior bladder wall till both the ostia become visible as two small, round hypoechogenic spots (Figure 3B). Rotating the probe toward the parasagittal plane, starting from one of the ostia, the distal ureter can be visualized.

Most of the time the lumen of the ureter is empty. Using a high-frequency probe with appropriated image-zooming, the passage of urine through the ureter can easily be evidenced as transient dilatation of the ureter lumen by anechogenic urine. The ureter wall thickness can be measured, and peristalsis is intermittently visible.

Using color- or power-Doppler, a brief urine jet can often be seen from the orifice of the ureter into the bladder. Although this might be of some help in finding the ureter for the less experienced sonographer, color imaging is not a requisite for proper visualization of the terminal ureters.

A feasibility study by Pateman et al. (2013) showed that the pelvic part of the ureters can be identified on transvaginal ultrasound in 96% of cases. The median time to visualise the left and the right ureter was 8 and 9 seconds respectively. Time to find the ureter was affected by the experience of the sonographer, but the overall visualisation rate was not. Aas-Eng et al. (2019) reported a learning curve of 50 scans for the detection of the distal part of ureters by transvaginal sonography.

The distance between the urethra and the ostia of the ureters depends on bladder filling. Typically, the ureteral orifice is about 2 to 3 cm from the urethra and both ureteral ostia are about 3cm from each other (Dubbins et al., 1981). Although some bladder filling might help the orientation, excessive bladder filling hampers proper visualization.

A ureteral calculus is visible on ultrasound as a small bright hyperechogenic lesion within the lumen of the ureter (Figure 4). Often some dilatation of the ureter proximal to the calculus is seen, as well as ureteral peristalsis.

In case 3, the dilatation of the proximal ureter was prominent and, in transverse section, could be mistaken for an adnexal cyst (Figure 1C).

If a rounded cystic structure is evidenced on ultrasound examination, the probe should always be turned over 90° to exclude a tubular structure. The differential diagnosis of an anechogenic or hypo- echogenic parauterine tubular structure includes a hydrosalpinx, a dilated vessel, a bowel loop and a hydroureter. A dilated ureter is well delineated, does not have persistent flow on color- or power Doppler examination and can be followed caudally till it enters the bladder wall and ends at the ostium of the ureter.

Our cases illustrate the importance of the sonographic evaluation of the anterior compartment including the bladder and the distal ureters in lower abdominal pain or abnormal bleeding. Common urological causes of lower abdominal pain in women are cystitis, urethritis, lithiasis, and urinary retention (Pages-Bouic et al., 2015; De Wey et al., 2019). Selective tenderness over the urethra or the trigonum are suspicious for uretritis and cystitis respectively. Ureterolithiasis can be diagnosed by direct visualization of the stone in the ureter lumen or indirectly by observing the dilatation of the ureter lumen proximal to the lithiasis and by the pain elicited by selective pressure on the ureter. Acute urinary retention is best evaluated by abdominal ultrasonography, because the dimensions of an overfull bladder extend beyond the maximal focal depth of the vaginal ultrasound probe.

The differential diagnosis of abnormal bleeding in elderly women includes, besides gynecological pathology, intestinal causes (e.g. rectum cancer, hemorroids) and urological conditions, such as carunculae of the urethra, hemorrhagic cystitis, bladder or renal cancer, lithiasis, stricture of the ureter or kidney disease (Abdel-fattah et al., 2004; Bolenz et al., 2018).

Although endometriosis was beyond the scope of this paper, we acknowledge the importance of including the assessment of the ureters during each systematic sonographic expert examination in the preoperative endometriosis setting (Guerriero et al., 2016). In case of ureterolithiasis, hydroureter or suspicion of deep endometriosis the ultrasonographic examination should be complemented with an abdominal scan of the kidneys to rule out hydronephrosis (Portis et al., 2001).

## Conclusion

A woman presenting with lower abdominal pain or abnormal blood loss is usually referred to a gynecological unit for a transvaginal ultrasound scan. Ultrasonography is a cheap and dynamic tool, not only to assess the gynecological organs, but also to easily identify and evaluate the bladder and pelvic part of the ureters.

Important pathology of the lower urinary tract causing symptoms mimicking gynecological pathology can be readily diagnosed by transvaginal ultrasound examination. The evaluation of the bladder and the ureters should therefore be part of the standard gynecological ultrasound investigation.
